# Family physicians’ diagnostic gut feelings are measurable: construct validation of a questionnaire

**DOI:** 10.1186/1471-2296-14-1

**Published:** 2013-01-02

**Authors:** Christiaan F Stolper, Margje WJ Van de Wiel, Henrica CW De Vet, Alexander LB Rutten, Paul Van Royen, Marloes A Van Bokhoven, Trudy Van der Weijden, Geert Jan Dinant

**Affiliations:** 1 Caphri School for Public Health and Primary Care, Department of Primary Medicine, Maastricht University, P.O. Box 616, Maastricht, 6200 MD, The Netherlands; 2 Faculty of Psychology & Neuroscience, Department of Work and Social Psychology, Maastricht University, Maastricht, The Netherlands; 3 EMGO Institute for Health and Care Research, Department of Epidemiology and Biostatistics, VU University Medical Center, Amsterdam, The Netherlands; 4 Institute for Intellectually Impaired, Tilburg, The Netherlands; 5 Faculty of Medicine and Health Sciences, Department of Primary and Interdisciplinary Care, University of Antwerp, Antwerp, Belgium

**Keywords:** Gut feelings, Family medicine, General practitioners, Diagnostic reasoning, Questionnaire, Construct validation, Medical decision-making, Medical problem-solving, Intuition, Clinical reasoning

## Abstract

**Background:**

Family physicians perceive that gut feelings, i.e. a ‘sense of reassurance’ or a ‘sense of alarm’, play a substantial role in diagnostic reasoning. A measuring instrument is desirable for further research. Our objective is to validate a questionnaire measuring the presence of gut feelings in diagnostic reasoning.

**Methods:**

We constructed 16 case vignettes from real practice situations and used the accompanying ‘sense of reassurance’ or the ‘sense of alarm’ as reference labels. Based on the results of an initial study (26 family physicians), we divided the case vignettes into a group involving a clear role for the sense of reassurance or the sense of alarm and a group involving an ambiguous role. 49 experienced family physicians evaluated each 10 vignettes using the questionnaire. Construct validity was assessed by testing hypotheses and an internal consistency procedure was performed.

**Results:**

As hypothesized we found that the correlations between the reference labels and corresponding items were high for the clear-case vignettes (0.59 – 0.72) and low for the ambiguous-case vignettes (0.08 – 0.23). The agreement between the classification in clear sense of reassurance, clear sense of alarm and ambiguous case vignettes as derived from the initial study and the study population’s judgments was substantial (Kappa = 0.62). Factor analysis showed one factor with opposites for sense of reassurance and sense of alarm items. The questionnaire’s internal consistency was high (0.91). We provided a linguistic validated English-language text of the questionnaire.

**Conclusions:**

The questionnaire appears to be valid. It enables quantitative research into the role of gut feelings and their diagnostic value in family physicians’ diagnostic reasoning.

## Background

Uncertainty about diagnosing is a typical characteristic of general practice [1,2]. Gut feelings and some important aspects of context knowledge, i.e. all a family physician knows about a patient apart from the signs and symptoms, are not discussed in traditional textbooks on medical decision-making, but often used by family physicians to support their diagnostic reasoning [3-5]. Chest pain, for instance, may indicate cardiac diseases, gastro-esophageal or pulmonary disorders, but can also be a sign of musculoskeletal or mental illnesses. Since it is impossible in a family medicine setting to define signs and symptoms that prove the presence of myocardial problems, [6] gut feelings may contribute to the family physician’s medical decision-making. In the case of chest pain such feelings appear to be rather accurate [7] and in the case of diagnosing serious infections in children, the family physician’s gut feeling ‘that something is wrong’ is in fact the best predictor among all signs and symptoms [8,9]. The gut feeling ‘this is not normal’ when a physician observes a child has proved to be a sign that makes the physician question the child’s well-being [10]. A qualitative study concluded that certain gut feelings, which could be referred to as a ‘sense of reassurance’ and a ‘sense of alarm’, play a substantial role in family physicians’ diagnostic reasoning [11]. A sense of reassurance means that a family physician feels secure about the further management and course of a patient’s problem, even though he/she may not be certain about the diagnosis: ‘everything fits in’. A sense of alarm implies that a family physician worries about a patient’s health status, even though he/she has found no specific indications yet; it is a sense of ‘there’s something wrong here’. The sense of alarm as a diagnostic tool has been taken seriously by disciplinary tribunals and is even regarded as an element of the professional standards for doctors [12]. Gut feelings are based on the interaction between patient information and a physician’s knowledge and experience, [13] and can be considered as a kind of skilled intuition [14]. Dual process theories may explain how physician’s analytical reasoning like the use of Bayes theorem and algorithmic decision tools, and their non-analytical reasoning like pattern recognition and gut feelings continually interact as two modes of knowing and thinking [13,15]. Affect defined as a feeling of ‘goodness’ or ‘badness’ guides the decision making process [16-19]. Cognitive neuroscience research provides support for the view that emotions are a vital component of the decision making process, helping people to thread their ways through the huge amount of information and knowledge [20-22].

In medicine, quantitative research into the actual contribution of gut feelings to physicians’ diagnostic processes is still lacking. Appropriate study designs assessing the diagnostic value of gut feelings, the significance of determinants like experience and context knowledge and the effects of teaching students about gut feelings need an instrument that will be able to determine the presence of gut feelings in diagnostic reasoning [23,24]. In nursing, some questionnaires have been developed to explore retrospectively the significance of gut feelings in diagnostic reasoning. These questionnaires, however, do not determine the presence or absence of gut feelings in actual clinical reasoning in real practice and have only been applied in research into nursing [25-30]. Moreover, they are not practicable in a general practice setting due to their extensiveness.

Therefore, we composed a short questionnaire that determines the presence of gut feelings in the context of family physicians’ diagnostic reasoning, based on consensus statements obtained in a Delphi procedure about the definition and content of gut feelings in general practice (see Table 1) [31]. The first 6 items in the questionnaire were directly derived from the consensus statements describing the sense of reassurance (item 1) and the sense of alarm (items 2–6) in family physicians’ diagnostic reasoning. The items are rated using a 5-point Likert scale ranging from completely disagree to completely agree. A final item (item 7) was added to assess whether a case vignette elicited a gut feeling (a sense of reassurance or a sense of alarm) or whether this was impossible for the respondent to say or was not applicable.

**Table 1:** Questionnaire based on the consensus statements

**Consensus statements**	**No.**	**Questionnaire items**
A ‘sense of reassurance’ means that a family physician feels secure about the further management and course of a patient’s problem, even though he/she may not be certain about the diagnosis: everything fits in.	1(SR)	I feel confident about my management plan and/or about the outcome: it all adds up.
A ‘sense of alarm’ implies that a family physician worries about a patient’s health status, even though he/she has found no specific indications yet; it is a sense of ‘there’s something wrong here’.	2(SA)	I am concerned about this patient’s state of health: something does not add up here.
A ‘sense of alarm’ activates the diagnostic process by stimulating a family physician to formulate and weigh up working hypotheses that might involve a serious outcome.	3(SA)	In this particular case, I will formulate provisional hypotheses with potentially serious outcomes and weigh them against each other.
A ‘sense of alarm’ means that a family physician perceives an uneasy feeling as he/she is concerned about a possible adverse outcome.	4(SA)	I have an uneasy feeling because I am worried about potentially unfavorable outcomes.
A ‘sense of alarm’ means that, if possible, the family physician needs to initiate specific management to prevent serious health problems.	5(SA)	This case requires specific management to prevent any further serious health problems.
	6(SA)	This patient’s situation gives me reason to arrange a follow-up visit sooner than usual or to refer him or her more quickly than usual to a specialist.
	7(FJ)	Please indicate what kind of gut feeling you had at the end of the consultation:
		* Something is wrong with this picture.
		* Everything fits.
		* Impossible to say, or not applicable.

Explanation of abbreviations: *SR* sense of reassurance, *SA* sense of alarm, *FJ* final judgment.

The objective of the present study was to assess the construct validity of this questionnaire in a group of experienced general practitioners and to determine its factor structure and internal consistency.

## Methods

### Study design

**Figure 1:**
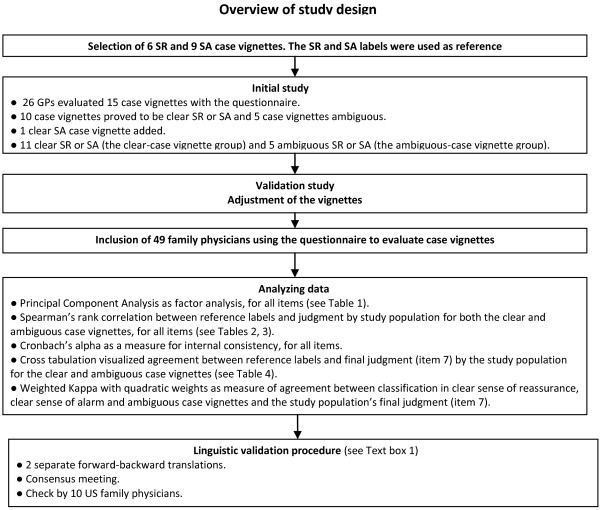
Explanation of abbreviations: SR= ‘sense of reassurance’, SA= ‘sense of alarm’.

Since there is no reference standard for the sense of reassurance and the sense of alarm, and no outcome measures of the concepts have been determined, it is not possible to assess criterion validity. Instead, we performed a construct validation procedure by testing whether the empirical data gathered with the questionnaire corresponded with the expectations based on the theoretical construct, i.e. the concept of gut feelings [32,33]. To this end, we constructed case vignettes from real practice situations in which gut feelings had played a role, and postulated hypotheses about the relations between the items of the questionnaire and the features of these case vignettes. Figure 1 provides a summary of the study design. The case vignettes can be regarded as an operationalization of the concept of gut feelings. The sense of reassurance or the sense of alarm, assigned to each of these cases was used as reference label.

### Selection of case vignettes

Based on real patient cases encountered by the 5 family physicians in the project group, we constructed vignettes of cases in which a sense of reassurance or a sense of alarm had played a role in the diagnostic approach of the attending family physician. After discussion, we accepted 15 case vignettes (5 sense of reassurance and 10 sense of alarm vignettes). Each case vignette had a format starting with the contextual information the family physician already has about the patient, followed by the patient’s complaints and finally additional information gathered during the encounter.

In an initial study, we asked 27 family physicians to evaluate the case vignettes by stating the most likely diagnosis and the treatment approach they would envision, and by completing the gut feelings questionnaire for each vignette. We also asked them to add their comments on the phrasing of the case vignettes. These family physicians participated in an earlier study of gut feelings as members of focus groups and were at the time selected by asking the teaching staff at three Departments of General Practice in the Netherlands for names of non-academic GPs interested in reflecting on diagnostic reasoning [11]. They equally represented both male and female, and experienced and inexperienced family physicians. Half of the participants received case vignettes numbers 1–8 and the other half numbers 8–15. Twenty-six family physicians (42% female, *mean number* of years of experience = 14, *SD* = 9) returned completed questionnaires. In 10 of the case vignettes, the sense of alarm (numbers 2, 4, 6, 10, 13, 14) and the sense of reassurance (numbers 1, 5, 8, 12) we expected based on our reference labels were recognized by most of the participants (64–100% agreement). In 5 of the case vignettes, the sense of alarm (numbers 3, 7, 15) and the sense of reassurance (numbers 9, 11) were not consistently recognized by the participants as intended (21–50% agreement). Based on these results, we distinguished between a group of vignettes featuring a clear role for gut feelings (‘clear-case’ vignettes) and a group with a more ambiguous role for gut feelings (‘ambiguous-case’ vignettes). Minor adaptations to the phrasing of the case vignettes were made, based on the comments of the 26 respondents. We also added a 16th illustrative case vignette of a patient visiting an out-of-hours medical service where the sense of alarm played a major role [34]. This case vignette (no. 16) and another one (no. 8) are presented in Table 2.

**Table 2:** Examples of case vignettes

**Case vignette 8 (sense of reassurance)**	
hi)	Patient is 34 years old and works as a sales assistant in a bakery. She is married with two children. Her medical history is uneventful. She does not smoke and drinks little alcohol. The only medication she is on is Microgynon 30 (oral contraception). No significant matters in the family history.
sy)	Patient visits her family physician because of a burning sensation she’s had for the last two days when urinating. She also feels pain and itch in her labias. She reports some discharge from the vulva. She was given amoxicillin for a lower airways infection two weeks ago. She has had the same complaints after a previous course of antibiotics, but cannot remember the name of the drug she was given then.
si)	Patient does not appear ill and has a normal complexion. External gynecological examination shows no abnormalities. There is vaginal discharge which resembles curdled milk. No further abnormalities are visible. Urine test strips for leukocytes, blood and nitrite are negative.
**Case vignette 16 (sense of alarm)**	
hi)	A 49-year-old woman phones the ‘triagist’ at an out-of-hours medical service at 21:40 h to report pain in her left side which has been increasing over the past 4 days. The pain is linked to her breathing and feels like sore muscles. The pain appears to be episodic to some extent; there are times when it is clearly less severe. The patient does not feel an urge to move. She currently has no pain elsewhere, and has had none during the past few days. Apart from a caesarian section ten years ago, she has no medical history. The only medication the patient is on is oral contraception. She has no known allergies. The triagist decides to invite her to visit the out-of-hours medical service post the same night.
sy)	As the physician collects her from the waiting room, the pain makes her walk with a stoop and she seems to experience shooting pains with each breath. She occasionally cries out for pain.
si)	Blood pressure is 128/84 mm Hg, pulse rate 90 a minute, regular and even. Saturation rate is 97%. Auscultation of the heart reveals no abnormalities and the lungs present vesicular breath sounds. A striking feature is the marked local tenderness of the musculature on the left side of the thorax. Calves are supple. A chest X-ray made within the past week shows no abnormalities. The family physician decides to administer an intramuscular injection of diclofenac 75 mg combined with 2 mg diazepam, which seems to be reasonably effective.

Explanation of abbreviations: hi= history, sy= symptoms, si= signs.

### Hypotheses

In accordance with the construct validation procedure, and assuming that the questionnaire could correctly distinguish between a sense of reassurance and a sense of alarm, we then drew up hypotheses about the questionnaire scores (see Table 3).

**Table 3:** Hypotheses

**First general hypothesis, relating to diagnostic characteristics with regard to clear-case vignettes:**	
The correlation between the reference labels of the clear-case vignettes (i.e. sense of reassurance or sense of alarm) and the answers given by the study population to questionnaire items reflecting a sense of reassurance or a sense of alarm is moderate to high.	
Specific hypotheses derived from this:	
a)	Comparing labels: there is a moderate to high correlation and agreement between the reference labels of the clear-case vignettes and the final sense of reassurance or sense of alarm judgment given by the study population (item 7).
b)	Comparing items of the study population: there is a moderate to high negative correlation between the sense of reassurance item (item 1) and the final sense of reassurance or sense of alarm judgment (item 7) for the clear-case vignettes. There is a moderate to high positive correlation between the sense of alarm items (items 2–6) and the final sense of reassurance or sense of alarm judgment (item 7) for the clear-case vignettes.
c)	Comparing items of the study population: there are moderate to high negative correlations between the sense of reassurance item (item 1) and the sense of alarm items (items 2–6) for the clear-case vignettes, whereas the intercorrelations between the sense of alarm items (items 2–6) are moderate to high.
**Second general hypothesis, relating to diagnostic characteristics with regard to the ambiguous-case vignettes:**	
The correlation between the reference labels of the ambiguous-case vignettes (i.e. sense of reassurance or sense of alarm) and the answers given by the study population to items reflecting a sense of reassurance or sense of alarm is weak to absent.	
Specific hypotheses derived from this:	
d)	Comparing correlations: there is a weaker correlation between the reference labels of the ambiguous-case vignettes and the final sense of reassurance or sense of alarm judgment (item 7) given by the study population compared to the clear-case vignettes.
e)	Comparing items of the study population: there is a weak correlation between the sense of reassurance item (item 1) or sense of alarm items (items 2–6) and the reference labels, but a moderate to high correlation between the sense of reassurance or sense of alarm items (items 1–6) and the final sense or reassurance or sense of alarm judgment (item 7).

### Validation study

Finally, we purposively selected 52 experienced family physicians from different regions in the Netherlands, as an earlier study had suggested that the level of experience is positively associated with the use of gut feelings [11] and invited them by phone to participate. To avoid selection bias, they were told that we were studying the diagnostic reasoning process of family physicians, without mentioning that the purpose of the study was to get information about gut feelings. Each of them received by post a different set of 10 case vignettes, selected so as to balance the number of clear and ambiguous cases, and varying the presentation order. We asked the participants to evaluate the vignettes by stating the most likely diagnosis and the treatment approach they envisioned, and to complete the questionnaire for each vignette. Forty-nine family physicians (63% female, *mean number* of years of experience = 20, *SD* = 9) of the 52 invited family physicians completed 10 questionnaires each. The reason to decline was lack of time.

### Statistical analyses

First, to examine whether the items concerning sense of alarm and sense of reassurance formed one or two dimensions, we examined the factor structure employing a Principal Component Analysis. The internal consistency was measured by calculating Cronbach’s alpha, after reversing the polarity of the sense of reassurance item (item 1) [35].

To examine the hypotheses in our construct validity procedures, we used Spearman’s rank correlations because of the categorical, ordinal nature of the data. We used the usual interpretation of the correlation values: weak < 0.3, moderate 0.3–0.6 and high > 0.6 [36].

We then used a cross tabulation to visualize the extent of agreement per vignette between the reference labels of the case vignettes and the final judgment (item 7) given by the study population. We also calculated a weighted Kappa using quadratic weights to assess the chance-adjusted agreement between the classification of the case vignettes (3 categories based on the results of the initial study: clear sense of reassurance; ambiguous; clear sense of alarm case vignettes) and the final judgment given by the study population in item 7 (3 categories: sense of reassurance, impossible to say or not applicable, sense of alarm). We used the following interpretation of the Kappa values: < 0.2 = slight, 0.2–0.4 = fair, 0.4–0.6 = moderate, 0.6–0.8 = substantial and > 0.8 = almost perfect [37]. Statistical calculations were performed using SPSS-16.

### Linguistic validation

For this publication and to allow a broad application of the Dutch-language questionnaire we translated the questionnaire into American-English using a formal linguistic validation procedure [38]. This involved two separate forward-backward translations, a consensus meeting and a check on cultural misunderstandings by ten US family physicians [39-41]. The text of the questionnaire is the result of this procedure (see Table 1) (see Endnote).

### Ethical approval

Since no patients were involved, this research did not fall under the Dutch Medical Research Involving Human Subjects Act (WMO) or the Embryos Act, so that no ethical permission was required.

## Results

The Principal Component Analysis showed one factor with the sense of reassurance and the sense of alarm items as two opposites, explaining 70.2% of total variance (see Table 4). The internal consistency of the gut feelings questionnaire was high (Cronbach’s alpha = 0.91).

**Table 4:** Factor analysis on items 1-7

**Component matrix**	
**Items**	**Component**
	1
1(SR)	-.722
2(SA)	.781
3(SA)	.924
4(SA)	.893
5(SA)	.809
6(SA)	.832
7(FJ)	.885

Explanation of abbreviations:

*SR* sense of reassurance, *SA* sense of alarm.

*FJ* final judgment of study population.

The first general hypothesis for construct validity, that there is a moderate to high correlation between the reference labels of the clear-case vignettes and the answers given by the 49 family physicians from the validation study to items reflecting the sense of reassurance (item 1) or the sense of alarm (item 2–6), was confirmed (see Table 5). We found a high correlation (0.72) between the reference labels and the final sense of reassurance or sense of alarm judgment (item 7). In addition, when comparing the answers given by the study population, we found a moderate negative correlation (−0.60) between the sense of reassurance item (item 1) and the final sense of reassurance or sense of alarm judgment (item 7). Table 5 shows that the correlations between the sense of alarm items (items 2–6) and the final sense of reassurance or sense of alarm judgment (item 7) were moderate to high (ranging from 0.59 to 0.79). Finally, Table 5 also shows moderate to high inverse correlation patterns for the sense of reassurance item (item 1) and the sense of alarm items (items 2–6) (ranging from −0.45 to −0.63), whereas the intercorrelations between the sense of alarm items (items 2–6) were moderate to high (ranging from 0.56 to 0.84).

**Table 5:** Correlations between questionnaire items for the group of ‘clear-case’ vignettes (n= 49 family physicians)

**Items**	**1(SR)**	**2(SA)**	**3(SA)**	**4(SA)**	**5(SA)**	**6(SA)**	**7(FJ)**
1(SR)							
2(SA)	-.60						
3(SA)	-.52	.70					
4(SA)	-.63	.84	.68				
5(SA)	-.45	.76	.62	.67			
6(SA)	-.55	.73	.56	.67	.65		
7(FJ)	-.60	.79	.59	.77	.65	.75	
SR/SA label	-.53	.67	.55	.63	.59	.62	.72

All significant at *p* ≤ .01.

Explanation of abbreviations: *SR* sense of reassurance, *SA* sense of alarm.

*FJ* final judgment of study population.

SR/SA label: the reference labeling.

The second general hypothesis, that the correlation between the reference labels of the ambiguous-case vignettes and the answers given by the study population to items reflecting a sense of reassurance or a sense of alarm was weak or absent, was also confirmed (see Table 6). We did indeed find that the correlation between the reference labels of the ambiguous-case vignettes and the final sense of reassurance or sense of alarm judgment given by the study population (item 7) was weaker compared to that for the clear-case vignettes (0.16 versus 0.72) (see Tables 5 and 6) and that the correlations between the reference labels and the sense of reassurance item (item 1) or the sense of alarm item (items 2–6) were absent (ranging from 0.05 to 0.24). We also found that the participants’ ratings of the items 1–6 were consistent with their final sense of reassurance or sense of alarm judgments (item 7), as indicated by moderate to high correlations between these ratings and the final judgments (see Table 6).

**Table 6:** Correlations between questionnaire items for the group of ‘ambiguous-case’ vignettes (n= 49 family physicians)

**Items**	**1(SR)**	**2(SA)**	**3(SA)**	**4(SA)**	**5(SA)**	**6(SA)**	**7(FJ)**
1(SR)							
2(SA)	-.48						
3(SA)	-.49	.54					
4(SA)	-.60	.76	.56				
5(SA)	-.33	.63	.38	.41			
6(SA)	-.39	.73	.51	.70	.52		
7(FJ)	-.54	.83	.46	.76	.54	.69	
SR/SA label	-.08	.24	.05	.14	.21	.20	.16

All significant at *p* ≤ .01.

Explanation of abbreviations: S*R* sense of reassurance, *SA* sense of alarm.

*FJ* final judgment of study population.

SR/SA label: the reference labeling.

A cross tabulation visualized per vignette the extent of agreement, between the reference labels of the case vignettes and the final sense of reassurance or sense of alarm judgment (item 7) given by the study population (see Table 7). The table clearly shows that for the clear case vignettes this agreement was substantial or almost perfect (ranging from 62% to 97%), apart from case vignette no. 12 for which agreement was moderate (46%). The results for the ambiguous-case vignettes, however, were mixed: for two (case vignettes (no. 9 and 15) the agreement with the reference labels was substantial (64% and 76%, respectively), whereas agreement was low to fair for the other three vignettes (13%, 25%, and 39%) (see Table 7). In the validation study the family physicians’ final judgment (item 7) thus differed from that of the initial study group on three case-vignettes (no. 9, 12 and 15). Still, the kappa with quadratic weighting showed that chance-adjusted agreement between the classification of the case vignettes as clear sense of reassurance, clear sense of alarm, or ambiguous case-vignettes based on the results of the initial study and the final judgment given in the validation study in item 7 (sense of reassurance, sense of alarm, impossible to say or not applicable) was substantial (0.62, 95% CI: 0.55-0.69).

**Table 7:** Number of participants of the study population that agreed upon the SR or SA reference label (% of agreement in bold) in their final judgment (item 7) for each vignette in both the ‘clear-case’ group and the ‘ambiguous’ group

**Case vignette**	**Reference label**	**N**	**Final judgment**		
			**SA**	**Impossible to say or not applicable**	**SR**
‘Clear-case’ vignettes					
no. 2	SA	29	25 **(86%)**	2	2
no. 4	SA	31	21 **(68%**)	8	2
no. 6	SA	32	23 (**72%)**	7	2
no. 10	SA	36	33 **(92%)**	1	2
no. 13	SA	32	26 **(81%)**	3	3
no. 14	SA	30	20 **(67%)**	7	3
no. 16	SA	25*	16 **(62%)**	5	4
no. 1	SR	29	1	1	27 **(93%)**
no. 5	SR	31	1	3	27 **(87%)**
no. 8	SR	32*	0	0	32 **(97%)**
no. 12	SR	26	10	4	12 **(46%)**
‘Ambiguous-case’ vignettes					
no. 3	SA	30	4 **(13%)**	8	18
no. 7	SA	36	9 **(25%)**	12	15
no. 15	SA	25	19 **(76%)**	4	2
no. 9	SR	35*	4	8	23 **(64%)**
no. 11	SR	28	11	6	11 **(39%)**

Explanation of abbreviations: *SR* sense of reassurance, *SA* sense of alarm, *one missing value.

## Discussion

The purpose of this study was to assess the construct validity of a questionnaire to measure gut feelings in general practice, which was based on consensus statements obtained in a Delphi procedure. The construct validity proved to be good. The correlation patterns were consistent with our hypotheses and there was a substantial agreement in judgments between the family physicians in the initial and the validation study. Factor analysis showed that the questionnaire clearly determined one gut feelings factor, with the sense of reassurance and the sense of alarm as two opposites, and the questionnaire had a high internal consistency. Our questionnaire to determine gut feelings in general practice thus passed an important validation test. The careful linguistic validation procedure makes it appropriate for research across countries. As far as we know, this is the first validated instrument providing a measure of the extent to which a sense of reassurance or a sense of alarm is present during the process of diagnosis. The results of the Delphi consensus procedure can be regarded as a sound basis for the content of the questionnaire. The successful construct validation procedure confirms that gut feelings play a role in family physicians’ diagnostic reasoning process, so now quantitative research can be started to examine their actual contribution.

We tried to compare our gut feelings questionnaire with comparable instruments by performing an English-language search in PubMed and Cinahl using the search terms ‘questionnaire’ [Mesh] OR ‘weights and measures’ [Mesh] AND ‘intuition’ [Mesh] OR ‘gut feelings’ OR ‘sense of reassurance’ OR ‘sense of alarm’. This search only yielded some extensive questionnaires examining the meaning of intuition in the field of nursing [25,26,28-30,42] retrospectively exploring not only the use of intuition and the extent of acknowledgment of intuition, but also other phenomena like physical sensations, premonitions, and reading of cues, cognitive behaviors, experience, skills and clinical thinking. In contrast, our gut feelings questionnaire only aims to determine the *presence* of a sense of reassurance and a sense of alarm in actual family physicians’ diagnostic reasoning, in observational as well in experimental research designs [24]. The questionnaire is short, easy to complete and feasible for use during routine office hours.

A limitation of our validation approach is that we used the outcomes of the diagnostic reasoning process of the 26 participants of the initial study as a reference to interpret the outcomes in the present validation study. Obviously, we needed such a procedure because there is no gold standard for gut feelings in general practice and we wanted to extend our reference beyond the agreement of the 5 family physicians who were part of the project group. The classification of case vignettes into a clear-case vignette group and an ambiguous one was consistent with actual practice with sometimes vague presentations of disease pictures and diagnostic uncertainty. It allowed us to hypothesize in which cases the correlations between the items in the questionnaire and the reference labels of the case vignettes (i.e., sense of reassurance or sense of alarm) would be moderate to high, or weak to absent. The hypothesized patterns were clearly confirmed by the data, although there were three case vignettes where the classification of the case vignettes as clear sense of reassurance, clear sense of alarm, or ambiguous case vignettes based on the initial study were not consistent with the judgment by the participants of the validation study. Case vignette 12 would now be classified as an ambiguous case, while cases vignettes 9 and 15 would now be classified as clear cases. The shift might be due to differences of experience between the family physicians participating in the initial and validation study and perhaps to the minor adaptations to the phrasing of the case vignettes made after the initial study.

Nevertheless, overall agreement in judgments between the 26 family physicians of the initial study and the 49 family physicians of the validation study was substantial. Moreover, when we based the classification (in further analysis) on the judgments of the 49 experienced family physicians the correlations were even higher for the clear-case vignettes and virtually absent for the ambiguous-case vignettes. We cannot expect full agreement, as gut feelings are based on the interaction between a family physician’s knowledge and experience and the patient information available [13]. As we represented real life situations in the case vignettes in our study, the participants could not further investigate their gut feelings by questioning or examining a patient. Research, therefore, should be extended to real practice situations where additional information can be obtained from patients.

After the validation, our questionnaire can now be used to start further research into the role of gut feelings in diagnostic reasoning, as well as research to assess the significance of the determinants and the effects of educational interventions related to gut feelings. The questionnaire may be useful for studies among specialists and nurses too. A European research agenda on gut feelings [24] gives the highest priority to questions about the prevalence of a sense of reassurance and a sense of alarm in routine practice and their diagnostic accuracy. Gut feelings may function as a compass in uncertain and complex situations, and now outcomes research may reveal to what extent this tool guides family physicians towards correct decisions and a proper diagnostic management and whether there are differences between experienced family physicians and trainees. Comparisons of situations in which family physicians manage patients they know and patients they see for the first time (e.g. when they are on call) may be used to assess the role of context knowledge. In terms of medical training, an interesting topic would be to study how students, trainee doctors and even experienced practitioners can be trained to develop, recognize and use gut feelings while avoiding pitfalls. In more experimental research, standardized patients or written clinical cases varying in the amount of contextual and patient knowledge could be presented for diagnosis to trainees and family physicians, to study the contribution of major diagnostic cues.

## Conclusions

Family physicians’ diagnostic gut feelings are measurable with the validated questionnaire. Diagnostic reasoning belongs to the core business of family physicians, and qualitative research has shown that gut feelings play a substantial part in this process. Our questionnaire should now make it possible to take the next crucial steps toward more quantitative research.

## Endnote

A British-English version has been obtained in a similar way and is available too (http://www.gutfeelingsingeneralpractice.eu).

## Competing interests

The authors declare that they have no competing interests.

## Authors’ contributions

Conception and design: CF Stolper, MWJ Van de Wiel, HCW de Vet. ALB Rutten, P Van Royen, MA Van Bokhoven, T Van der Weijden, GJ Dinant. Analysis and interpretation of the data: CF Stolper, MWJ Van de Wiel, HCW de Vet, ALB Rutten. Drafting of the article: CF Stolper, MWJ Van de Wiel, HCW de Vet. Critical revision of the article for important intellectual content: P Van Royen, MA Van Bokhoven, T Van der Weijden, GJ Dinant. Statistical expertise: ALB Rutten, HCW de Vet. All authors read and approved the final manuscript.

## Pre-publication history

The pre-publication history for this paper can be accessed here:

http://www.biomedcentral.com/1471-2296/14/1/prepub

## References

[B1] Dinant GJ Jones R, Britten N, Gulpepper L, Gass D, Grol R, Mant D Diagnosis and decision. Undifferentiated illness and uncertainty in diagnosis and management Oxford Textbook of Primary Medical Care 2004 Oxford: Oxford University Press 201 203

[B2] Griffiths F Green E Tsouroufli M The nature of medical evidence and its inherent uncertainty for the clinical consultation: qualitative study BMJ 2005 330 511 517 10.1136/bmj.38336.482720.8F 15684026 PMC552810

[B3] Bruyninckx R Van den Bruel A Hannes K Buntinx F Aertgeerts B GPs' reasons for referral of patients with chest pain: a qualitative study BMC Fam Pract 2009 10 55 10.1186/1471-2296-10-55 19646225 PMC2731044

[B4] Hani MA Keller H Vandenesch J Sonnichsen AC Griffiths F Donner-Banzhoff N Different from what the textbooks say: how GPs diagnose coronary heart disease Fam Pract 2007 24 622 627 10.1093/fampra/cmm053 17971349

[B5] Johansen ML Holtedahl KA Rudebeck CE How does the thought of cancer arise in a general practice consultation? Interviews with GPs Scand J Prim Health Care 2012 30 135 140 10.3109/02813432.2012.688701 22747066 PMC3443936

[B6] Bruyninckx R Aertgeerts B Bruyninckx P Buntinx F Signs and symptoms in diagnosing acute myocardial infarction and acute coronary syndrome: a diagnostic meta-analysis Br J Gen Pract 2008 58 105 111 18307844 10.3399/bjgp08X277014 PMC2233977

[B7] Buntinx F Truyen J Embrechts P Moreel G Peeters R Chest pain: an evaluation of the initial diagnosis made by 25 Flemish general practitioners Fam Pract 1991 8 121 124 10.1093/fampra/8.2.121 1874355

[B8] Van den Bruel A Haj-Hassan T Thompson M Buntinx F Mant D Diagnostic value of clinical features at presentation to identify serious infection in children in developed countries: a systematic review Lancet 2010 375 834 845 10.1016/S0140-6736(09)62000-6 20132979

[B9] Van den Bruel A Aertgeerts B Bruyninckx R Aerts M Buntinx F Signs and symptoms for diagnosis of serious infections in children: a prospective study in primary care Br J Gen Pract 2007 57 538 546 17727746 PMC2099636

[B10] Lykke K Christensen P Reventlow S "This is not normal … "–signs that make the GP question the child's well-being Fam Pract 2008 25 146 153 10.1093/fampra/cmn021 18515812

[B11] Stolper CF Van Bokhoven MA Houben PHH Van Royen P Van de Wiel M Van der Weijden T The diagnostic role of gut feelings in general practice. A focus group study of the concept and its determinants BMC Fam Pract 2009 10 17 10.1186/1471-2296-10-17 19226455 PMC2649045

[B12] Stolper CF Legemaate J Dinant GJ How do disciplinary tribunals judge the 'gut feelings' of doctors? An analysis of Dutch tribunal decisions 2000-2008 J Law and Medicine 2010 18 68 75 20977163

[B13] Stolper CF Van de Wiel M Van Royen P Van Bokhoven MA Van der Weijden T Dinant GJ Gut feelings as a third track in general practitioners' diagnostic reasoning J Gen Intern Med 2011 26 197 203 10.1007/s11606-010-1524-5 20967509 PMC3019314

[B14] Kahneman D Klein G Conditions for intuitive expertise: a failure to disagree Am Psychol 2009 64 515 526 19739881 10.1037/a0016755

[B15] Pelaccia T Tardif J Triby E Charlin B An analysis of clinical reasoning through a recent and comprehensive approach: the dual-process theory 2011 Online: Med Educ 16 10.3402/meo.v16i0.5890 PMC3060310 21430797

[B16] Slovic P Finucane M Peters E MacGregor DG Gilovich T, Griffin D, Kahneman D The Affect Heuristic Heuristics and biases 2002 New York: Cambridge University Press 397 420

[B17] Finucane M Peters E Slovic P Schneider SL, Shanteau J Judgement and decision making: The dance of affect and reason Emerging Perspectives on Judgement and Decision Research 2003 Cambridge, UK: Cambridge University Press 327 364

[B18] Epstein S Integration of the cognitive and the psychodynamic unconscious Am Psychol 1994 49 709 724 8092614 10.1037//0003-066x.49.8.709

[B19] Mikels JA Maglio SJ Reed AE Kaplowitz LJ Should I go with my gut? Investigating the benefits of emotion-focused decision making Emotion 2011 11 743 753 21639628 10.1037/a0023986

[B20] Craig AD How do you feel–now? The anterior insula and human awareness Nat Rev Neurosci 2009 10 59 70 10.1038/nrn2555 19096369

[B21] Naqvi N Shiv B Bechara A The Role of Emotions in Decision Making: a Cognitive Neuroscience Perspective Curr Dir Psychol Sci 2006 15 260 264 10.1111/j.1467-8721.2006.00448.x

[B22] Damasio AR Descartes' Error: Emotion, Reason, and the Human Brain 1994 New York: Avon

[B23] Buntinx F Mant D Van den Bruel A Donner-Banzhof N Dinant GJ Dealing with low-incidence serious diseases in general practice Br J Gen Pract 2011 61 43 46 10.3399/bjgp11X548974 21401991 PMC3020049

[B24] Stolper CF Van Leeuwen Y Van Royen P Van de Wiel M van Bokhoven MA Houben PHH Establishing an international research agenda on gut feelings in general practice using the nominal group technique Eur J of Gen Pract 2010 2 75 79 20192888 10.3109/13814781003653416

[B25] Smith AJ The development and psychometric testing of an instrument measuring the use of intuition by nursing students 2003 University of San Diego: Dissertation

[B26] Smith A Measuring the use of intuition by registered nurses in clinical practice Nurs Stand 2007 21 35 41 10.1007/BF03079502 17824453

[B27] Smith AJ Thurkettle MA dela Cruz FA Use of intuition by nursing students: instrument development and testing J Adv Nurs 2004 47 614 622 10.1111/j.1365-2648.2004.03149.x 15324430

[B28] Miller VG Measurement of self-perception of intuitiveness. West J Nurs Res 1993 15 595 606 10.1177/019394599301500506 8236960

[B29] Rew L Acknowledging intuition in clinical decision making J Holist Nurs 2000 18 94 13 10.1177/089801010001800202 11847771

[B30] Miller VG The development of an instrument to measure self-perception of intuitiveness of practicing nurses 1990 University of Texas at Austin: Dissertation

[B31] Stolper CF Van Royen P Van Bokhoven MA Houben PHH Van de Wiel M Van der Weijden T Consensus on gut feelings in general practice BMC Family Practice 2009 10 66 10.1186/1471-2296-10-66 19761589 PMC2754436

[B32] Smith GT On Construct Validity: Issues of Method and Measurement Psychol Assess 2005 17 396 408 16393005 10.1037/1040-3590.17.4.396

[B33] Vet de HCW Terwee CW Mokkink LB Knol DL Measurement in Medicine. A practical guide 2011 Cambridge: Cambridge University Press

[B34] Baks M Pulmonary embolism often not immediately evident [Longembolie lang niet altijd direct duidelijk] Huisarts 24/7 2009 28 29

[B35] Bland JM Altman DG Cronbach's alpha BMJ 1997 314 572 10.1136/bmj.314.7080.572 9055718 PMC2126061

[B36] Cohen J The significance of a product moment r Statistical power analysis for the behavioural sciences 1988 Hillsdale, New Yersey: Erlbaum 75 107

[B37] Landis JR Koch GG The measurement of observer agreement for categorical data Biometrics 1978 33 159 174 843571

[B38] MAPI Institute http://www.mapi-institute.com/linguistic-validation. last seen 21-12-2012.

[B39] Acquadro C Conway K Hareendran A Aaronson N Literature review of methods to translate health-related quality of life questionnaires for use in multinational clinical trials Value Health 2008 11 509 521 10.1111/j.1524-4733.2007.00292.x 18179659

[B40] Streiner DL Norman GR Health Measurement Scales. A practical guide to their development and use 2006 3 Oxford: Oxford University Press

[B41] Acquadro C Conway K Giroudet C Mear I Linguistic validation manual for patient-reported outcomes (PRO) instruments 2004 Lyon: MAPI Research Institute

[B42] Cone KJ The development and testing of an instrument to measure decision making in emergency department triage nurses 2000 Saint Louis University: Dissertation

